# The Cave Report

**Published:** 1921-06-11

**Authors:** 


### The Hospital

The Workers' Newspaper of Administrative Medicine and Institutional Life, Administration, National Insurance and Health.

No. 1827, Vol. LXX. SATURDAY, JUNE 11, 1921. PRICE TWOPENCE

### THE CAVE REPORT.

A summary of the recommendations of the Voluntary Hospitals Committee will be found on page 174. The Report in its entirety will be issued a little later, and until it is possible to read the two documents side by side it is not easy completely to grasp the full meaning of the recommendations. The Report, it is understood, divides itself into two parts; one deals with suggestions to hospitals on matters which are already under their own control, the other is descriptive of proposed changes which will probably require legislation.

The first group of suggestions includes such things as reduction of expenditure, co-operative buying, appeals for donations, contributions by wage-earners and employers, other contributory schemes, and contributions by Approved Societies. The second group embraces many forms of assistance of greater or lesser importance.

It is proposed that a Hospitals Commission and local voluntary hospitals committees be formed. King Edward's Hospital Fund for London is nominated for the latter service throughout the Metropolitan Police District. Parliament, it is proposed, is to be asked to grant £1,000,000 to be expended under the direction of the Hospitals Commission for assisting needy hospitals. Thus far the details of the Report that have leaked out are confirmed. Amongst the points that are new, one of the chief is that the Commission is authorised during two years to recommend grants (on the "pound-for-pound" system) for extension and improvement. This explains, to some extent, the seeming inadequacy of the million pound grant, for that sum would hardly be sufficient to cover arrears in maintenance and improvement of buildings, let alone provide the more urgent requirements for current expenses of a general nature.

The lesser recommendations deal with extended use of infirmaries by Poor-law Guardians, contributions from county councils, payment for treatment of employees of local authorities, grants for nurse-training, deductions for income-tax purposes by employers who contribute, and remission of legacy and succession duty. These it is not possible at this stage more than to mention, although each is important. The predominant note in the Report is that the Voluntary System is sound at the core, it is passing through a crisis which it is possible to avert by a very moderate amount of help from public funds, and that, given that assistance and the whole-hearted help of the public, the system will emerge from its troubles greater than ever.

